# What Are LGBT+ Inequalities in Health and Social Support—Why Should We Tackle Them?

**DOI:** 10.3390/ijerph18073612

**Published:** 2021-03-31

**Authors:** Julie Fish, Kathryn Almack, Trish Hafford-Letchfield, Michael Toze

**Affiliations:** 1 Social Work and Health Inequalities, School of Applied Social Sciences, De Montfort University, Leicester LE1 9BH, UK; 2 School of Health and Social Work, University of Hertfordshire, Hatfield, Hertfordshire AL10 9EU, UK; k.almack@herts.ac.uk; 3 School of Social Policy and Social Work, School of Humanities and Social Sciences, University of Strathclyde, 16 Richmond Street, Glasgow G1 1XQ, UK; trish.hafford-letchfield@strath.ac.uk; 4 Public Health and Social Determinants of Health, Lincoln Medical School, University of Lincoln, Brayford Pool, Lincoln LN6 7TS, UK; mtoze@lincoln.ac.uk

## 1. Introduction

Health inequalities are differences in health experiences and outcomes which arise through the everyday circumstances of people’s lives and the appropriateness of the systems put in place to support them. Such differences stem from social inequalities. As they can be alleviated through social policy, they are a key concern for global public health. Worldwide, they are the focus of governmental efforts to reduce avoidable differences in health (e.g., in the UK, Health equity in England 2020 [1] and in the USA, Healthy People 2030 [2]). The salience of health inequalities in public life cannot be over-emphasized; for example, it has informed policymaking since Engels’ [3] 1845 ground-breaking text on the condition of the working class in England and numerous policy initiatives since then (e.g., in the UK, The Black Report 1979, The Acheson Inquiry 1998, Fair Society, Healthy Lives 2010).

In an international context, the World Health Organization (WHO) [4] has developed an equity framework for the Social Determinants of Health which identifies structural determinants including the socio-economic context, the processes of government, public policies, cultural and social value together with people’s protected characteristics. However, while gender, social class and ethnicity are acknowledged as influences on people’s circumstances, living conditions, health behaviors and psychosocial well-being; the influence of Sexual Orientation and Gender Identity (SOGI) on health equity for LGBT+ people is overlooked. This contributes to the subordinate status of SOGI in international policymaking, practice developments and the funding of research to inform evidence-based decision-making.

There are a number of obstacles to the inclusion of research in LGBT+ health inequalities and social support in national and international policy initiatives including attitudinal (ranging from disbelief that differences exist to discriminatory views [5]), the relative dearth of robust data (due to lack of measures about SOGI in existing data-sets), and the lack of large-scale data-sets (due to challenges in random sampling). Of concern at a global level, is the unequal provision of rights for LGBT+ people. In six UN member states, the death penalty can be imposed for consensual same-sex sexual behavior and in 26 countries a penalty of 10 years to life imprisonment can be imposed [6,7]. Some jurisdictions separately penalize diverse gender expression and many countries have no framework for allowing trans people to access ID documents or state recognition; further, they impose requirements that are a breach of human rights, such as sterilization (https://www.humandignitytrust.org; accessed 30 March 2021). The Office of the High Commissioner for Human Rights (OHCHR) has noted the violation of the human rights of intersex people in many jurisdictions, e.g., non-urgent sterilizing surgeries performed on children without capacity to consent [8,9]. By contrast, protections for sexual orientation and gender identity are explicitly included by nine jurisdictions; moreover, in the constitutions of 123 UN member states consensual same sex acts are legal. Several international jurisdictions have implemented legislative ordinances to protect the rights of their LGBT+ citizens, there is increasing recognition by governments of the need to understand and address LGBT+ health and social care inequalities [10,11,12,13] and a number are implementing policy initiatives to promote well-being including adoption, equal marriage, protections in employment, housing and access to and use of health and social care. Globally, (although important omissions remain) there are legal frameworks to support the implementation of evidence-based policy to embed these legislative developments in everyday life. In this Special Issue, we aim to contribute to the substantive field of LGBT+ research, highlight methodological developments and new approaches to evaluation and implementation to support the work of Governmental and Non-Governmental Organizations (NGOs) and other bodies across the world in their LGBT+ equality work.

The USA has so far contributed the largest body of work in LGBT+ inequalities in health and social support [14]. In this Special Issue, we sought to include studies from other countries to reflect their growing contribution to these debates. In our call for papers, we aimed to give voice to those under-represented in LGBT+ research and the collection includes scholarship about the needs and experiences of LGBT+ people who are older, disabled, trans and gender diverse people, people with disabilities and intersex people. The Special Issue aims to shed light on the mechanisms which link SOGI with health and well-being to promote evidence-based policy and to frame initiatives for their implementation.

This Special Issue is partly shaped by a recent agenda-setting paper [15] which called for greater and more robust data to inform international policy developments. To this end, the authors advocated seven criteria on which to build a robust field of research to support international and national government initiatives aimed at tackling LGBT+ health inequalities. We endeavored to include studies which meet these criteria in the following ways: (1) large datasets (Li, Hickson, Kattari, Tan); (2) comparative data collection (Hickson); (3) addressing diversity and intersectionality among LGBT+ people (Daly, Hickson, Levin, Kattari, O’Shea, Toze); (4) investigating the delivery of healthcare services capacity to deliver LGBT+ affirmative healthcare (Levin); (5) identification of effective health promotion and/or treatment interventions and for sub-groups (Li, Toze); (6) development of a health equity model (Daly, Henrickson); (7) utilization of social justice concepts to ensure change-oriented data (Kattari, Mulé, McDermott).

## 2. Note on Terminology

Although we used the term LGBT+ in our call for papers, we did not require contributors to adopt this concept. Instead we encouraged the use of context specific terminology that are reflective of the ways in which study participants described themselves. As Henrickson et al. (this Special Issue) emphasize, scholars should use the taxonomy and language of communities, rather than requiring them to fit into pre-existing categories. For example, Two Spirit denotes indigenous peoples in Canada and the USA; while in New Zealand, the terms whakawahine, takat¯apui, and tangata ira t¯ane are used to signify gender diversity. Among young people, there is increasing use of the concept of Queer and three of the papers include this terminology.

## 3. Overview of Papers

Diana Kwok’s research in Hong Kong, China was conducted in a social and political context which lacks discrimination protection for LGBQ people. In an environment where sexual prejudice prevails, she undertook in-depth interviews with 67 social workers to understand their perspectives on prejudice reduction training and perceptions of cultural barriers. Emerging findings suggest that social workers and service providers need to understand how sexual prejudice is manifested in Hong Kong. The study recommendations include: introducing LGBQ curricula content, updating conceptual frameworks and the use of inclusive language in qualifying training. Intergroup contact, critical reflection and experiential learning are proposed strategies to enhance learning.

Andrea Daley et al.’s paper draws on key findings from the home care access project. The team conducted a mixed methods study across Ontario, Canada to understand the relevance and effectiveness of an access and equity framework to support the assessment, evaluation and implementation of access and equity strategies for 2SLGBTQ+ people. The framework includes six indicators of access to care: community engagement, leadership, environment, policies and processes, education and training, and programs and services.

Dian-Jeng Li et al.’s study investigated the history of homophobic bullying and experiences of sedative/hypnotic use alongside perceived family support and emotional symptoms of 500 Gay and Bisexual (aged 20–25) men in Taiwan. The findings revealed that a higher level of homophobic bullying was significantly associated with sedative/hypnotic use among gay and bisexual men. A higher level of family support protected gay and bisexual men who had experienced homophobic bullying in their childhood and adolescence from later sedative/hypnotic use. The study recommended that mental health professionals should treat the emotional symptoms of gay and bisexual men who have had previous experiences of homophobic bullying. The authors call for the establishment of an LGBT-friendly environment in school and timely interventions to prevent the emergence of mental health problems.

Mark Henrickson et al.’s review article proposes the Montreal 12 principles for ethical research with Gender and Sexually Diverse Persons and Communities. The authors urge that all those engaged in the research enterprise make an ongoing commitment to research that is inclusive, dynamic, and responsive to evolving language, communities, and expressions of gender and sexual diversity. Researchers can then provide sound evidence on which to base policy and interventions to address health and social inequities for all persons, and particularly for gender and sexually diverse persons.

Ford Hickson et al.’s study on the European MSM internet survey investigated sexual and mental health inequalities among 125,720 men living in 45 European and neighboring countries. The sample comprised of men who were assigned female at birth and/or identify as trans men. Anxiety, depression, alcohol dependence and sexual unhappiness were more prevalent in sex/gender minority men. Conversely HIV and STI diagnoses were less common. Sex assigned at birth and trans identification were associated with different sexual and mental health needs. To facilitate service planning and to foster inclusion, sex-assigned-at-birth and current gender identity should be routinely collected in health surveys.

Nina Levin Jackson et al.’s study is located within a canonical body of work, families we choose, conceptualized by US anthropologist Kath Weston. This current study used the terminology of chosen family to consider social support and the mutual provision of care in the context of health, care and wellbeing amongst queer and transgender young adults. The findings highlighted the ways participants provided advocacy in medical contexts, emergency contacts, organizing around health needs, assisting one another through changes in relationship contexts; sharing material resources and providing support.

Shanna Kattari et al.’s paper reports on findings from the Michigan Trans Health Survey which reports on mental health differences within trans communities. The survey revealed high rates of mental health concerns including depression, anxiety, non-suicidal self-injury thoughts, suicidal thoughts, engagement in non-suicidal self-injury and suicide attempts. There were increased rates of mental health disparities among those with disabilities. The study concludes that practitioners need to be aware of the mental health concerns in these communities and work across policy, interpersonal practice, and systems to support their clients’ mental health.

The article by Kyle Tan et al. examines differences in mental health inequities between younger and older transgender people and the general population of New Zealand finding significant differences. Trans women were less likely to report psychological stress than trans men and non-binary people. Moreover, older trans women were more likely to report psychological distress than younger trans women. They call for the introduction of inclusive practices to improve mental health and well-being for gender minority populations.

Nick J. Mulé takes a critical content analysis premised on queer liberation theory of Canada’s federal government’s report on LGBT+ health. The author finds that it presents an opportunity for a state response in areas including research, education, policy, funding and programming. While the report addresses the breadth of health concerns faced by Canada’s LGBT+ populations which has moved beyond the focus of HIV/AIDS, there is an undue emphasis on entry level recommendations and consequently the report offers a less progressive approach to address these issues.

Amie O’Shea et al. conducted a focus group study with peer researchers and takes an intersectional approach to understanding disabled LGBTIQA+ people’s access to support services in Victoria Australia. Two themes were identified: experiences of accessing health services as LGBTIQA+ people with a disability; difficulties in managing multiple identities and the impacts of community services and supports. Their research suggests that inclusive practices may be partly achieved through shifts in policy and practice that identifies specific needs of LGBTIQA+ people with a disability, LGBTIQA+ education for disability services and disability education strategies for LGBTIQA+ focused services.

Michael Toze et al.’s article addresses the lack of surveillance data in monitoring SOGI. In 2019, the UK Department of Health introduced a standard measure for asking about sexual orientation, but there continues to be uncertainty about its purpose and relevance. By applying a health capabilities perspective, the benefits and risks of disclosure can be mapped against multiple domains of capability. Disclosure is relevant not only for the individual concerned but also at a population level to better capture health inequalities across and within communities.

Laetitia Zeeman and Kay Aranda conducted a systematic review of intersex health and healthcare inequalities. Using the procedures set out in PRISMA, sixteen studies were included. People with intersex variance experience a higher incidence of anxiety, depression and psychological distress compared to the general population linked to stigma and discrimination. Findings support rethinking sex and gender to reflect greater diversity within a more nuanced sex-gender spectrum. More large-scale research, with the involvement of peer researchers, is needed to ensure policy, education and healthcare advances with greater inclusivity and ethical accountability.

Liz McDermott and Rosie Nelson have conducted a scoping review in cancer, mental health, and palliative care. They engaged with key stakeholders to identify research and policy priorities to inform public health interventions which address inequalities in LGBT+ health and social support. They propose three recommendations: developing large datasets with representative samples, ensuring that research disaggregates identities to examine differential health experiences within the category LGBT+, and utilising theories derived from social models of health to capture the complex cultural, social, economic and political factors that shape health inequalities for LGBT+ communities.

## 4. Concluding Remarks

This Special Issue contributes to understanding the social conditions in the lives of LGBT+ people which lead to health inequalities. The rationale for addressing them is to promote LGBT+ equality and human rights in health. Studies have explored the experiences of groups that have historically been under-researched including LGBT+ people with disabilities, indigenous peoples, trans, gender diverse and intersex people enabling researchers and policymakers to understand the heterogeneity within LGBT+ communities. In the endeavor to ensure that studies are reflective of communities’ perspectives and concerns, researchers have sought to involve peer researchers in the governance and conduct of studies. Current tools for evaluating the effectiveness of services often overlook LGBT+ people; studies in this volume have identified access and equity frameworks to support the evaluation and implementation of public health strategies for LGBT+ people. We believe that the collection can inform policy development and implementation across a range of issues in health and social support for diverse LGBT+ communities.
